# The Effect of Rod-Shaped Long-Period Stacking Ordered Phases Evolution on Corrosion Behavior of Mg_95.33_Zn_2_Y_2.67_ Alloy

**DOI:** 10.3390/ma11050815

**Published:** 2018-05-16

**Authors:** Jingfeng Wang, Weiyan Jiang, Shengfeng Guo, Yang Li, Yao Ma

**Affiliations:** 1 National Engineering Research Center for Magnesium Alloys, College of Materials Science and Engineering, Chongqing University, Chongqing 400044, China; jwy1020@126.com (W.J.); jwy1020@163.com (Y.L.); 15671628464@163.com (Y.M.); 2 State Key Laboratory of Mechanical Transmission, Chongqing University, Chongqing 400044, China; 3 Faculty of Materials and Energy, Southwest University, Chongqing 400715, China

**Keywords:** magnesium alloy, morphology evolution, corrosion behavior

## Abstract

The morphology evolution of long-period stacking ordered (LPSO) phases on corrosion behavior of Mg_95.33_Zn_2_Y_2.67_ alloy is investigated systematically during as-cast, pre-extrusion heat-treated, as-extruded and post-extrusion heat-treated conditions. The second phases in the as-cast alloy are only LPSO phases with a few Y particles. The pre-extrusion heat treatment changed LPSO phases from blocks into a rudimentary rod shape with lamellar structure, subsequently into fine fragments by extrusion, and then into a regular rod shape with lamellar structure followed by post-extrusion heat treatment. Immersion tests and electrochemical measurements in 3.5 wt % NaCl solution reveal that the post-extrusion heat-treated alloy has the best corrosion resistance with the lowest corrosion rate. This is attributed to the rod-shaped LPSO phases, which could hinder corrosion proceeding, and result in corrosion orientated along the direction of rods and forming relatively dense long-strip corrosion products. Our findings demonstrate that the improved corrosion resistance of magnesium alloys with LPSO phases can be tailored effectively by the proceeding technology and post-heat treatment.

## 1. Introduction

Combined with high specific strength, specific stiffness and excellent casting performance, magnesium and its alloys serve as the lightest structural material, and have become the focus of materials research [1,2]. In particular, magnesium alloy with long-period stacking ordered (LPSO) phases exhibit relatively high strength and good ductility, making them promising candidates for practical applications [3,4,5,6]. Unfortunately, their corresponding corrosion resistance is still a critical factor in restricting their utility. Magnesium alloys with LPSO phases will promote the activity of galvanic microcells, which usually results in a detrimental effect on the corrosion resistance of these alloys [7]. Extensive efforts have been devoted to elucidating the correlation between LPSO phases and corrosion resistance of magnesium alloys [8,9].

It was reported that an 18R LPSO structure formed in Mg–Zn–Y during solidification, while a 14H LPSO structure formed under the constant temperature in Mg–Zn–Gd [10]. R. Schmid-Fetzer et al. [11] confirmed this rule, and further revealed that the existence temperature area of the 18R LPSO structure was 753~483 °C, while the formation temperature of the 14H LPSO structure was lower than 537 °C. The LPSO phase [12] was found to be absent in the as-cast ingots but precipitated during soaking at 500 °C in Mg–Gd–Zn alloys. Zhang [13] reported that as-extruded Mg–11.3Gd–2.5Zn–0.7Zr (wt %) alloy with a lamellar LPSO phase exhibited better corrosion resistance and more uniform corrosion than Mg–10.2Gd–3.3Y–0.6Zr (wt %) alloy, wherein the LPSO phase was absent. By contrast, it was revealed by [14] that the lamellar LPSO structure along the grain boundary in as-cast Mg–5Gd–1Zn–0.6Zr (wt %) alloy can act as a barrier between the substrate and the eutectic phase and inhibit galvanic corrosion. In the Mg–Zn–Y alloys, the LPSO phase of 18R structure was obtained in the cast state which usually appeared in the crystal boundary, and turned to a 14H structure after heat treatment, which appeared laminated in the crystal. The corrosion rates of the as-cast Mg–Zn–Y alloys was a function of the volume fraction of LPSO phases due to their nobler nature than that of α-Mg matrix [15]. Most corrosion pits preferentially formed at the LPSO-phase/α-Mg interface and gradually developed along the direction of the LPSO phases. It was noted by [16] that the stated cooling rate played a key role in regulating the corrosion behavior of Mg–Zn–Y alloys with LPSO because of grain refinement and the formation of a supersaturated single-phase solid solution. However, there were still many controversies about the influence of LPSO phases on the corrosion progress of Mg–Zn–Y alloys. Pérez et al. [17] claimed that the corrosion rate of transversal sections of Mg_97_Y_2_Zn_1_ alloys was lower than that of their longitudinal counterparts in the long-term corrosion processes. Peripheral habits of the LPSO phases with respect to grains were desired to perform better corrosion resistance and a more uniform corrosion mode than those scattered throughout the whole grains [18]. The spatial arrangements of LPSO phases changed by the processing route and chemical composition also imposed a critical influence on the corrosion behavior of extruded Mg_97_Y_2_Zn_1_ alloy. When the LPSO phase was compact and thick, this could hinder the corrosion progress to a small degree. Therefore, the LPSO phases exhibited various types, implying the morphology, volume fractions and distribution of LPSO phases played a critical role in determining the corrosion behavior of Mg–Zn–Y alloys [19,20]. However, there is no specific study on how to control structure morphology and the distribution state of the LPSO phase and its effect on the corrosion properties of alloys. The underlying mechanism of how these factors influence the corrosion resistance and to what extent affect the corrosion resistance of magnesium alloy is still not fully understood. Therefore, this current work attempts to study the morphology control process of LPSO phases, obtain the corrosion behavior of the alloy, and explore the influence of LPSO phase morphology on the corrosion behavior of Mg–Zn–Y alloys.

In this study, as-cast, pre-extrusion heat-treated, as-extruded and post-extrusion heat-treated Mg_95.33_Zn_2_Y_2.67_ alloys were prepared, of which the LPSO phase precipitated with morphology and distribution changed significantly under different treatments. The corrosion behavior of these alloys are investigated systematically with the evolution of LPSO phases. In particular, the relation between the morphology of LPSO phases and the corresponding corrosion behavior of the alloys is revealed.

## 2. Materials and Methods

### 2.1. Materials Preparation

An as-cast Mg_95.33_Zn_2_Y_2.67_ alloy ingot was prepared in an induction furnace (Semi-continuous vacuum induction melting furnace, Jinzhou Zhongzhen Electric Furnace Co. Ltd., Jinzhou, China) under Ar atmosphere and using high-purity Mg (99.95 wt %), Zn (99.99 wt %), and Mg–28 wt % Y master alloy (Yueyang Yuhang New Material Co. Ltd., Yueyang, China). Pre-extrusion heat treatments were carried out at 540 °C for 5 h and then quenched with water at room temperature. Extrusions were carried out at 400 °C with an extrusion ratio of 11, and followed by post-extrusion heat treatment at 540 °C for 5 h.

### 2.2. Microstructural Analysis

Specimens for microstructural observation were polished and etched with a solution of the saturated picric acid, acetic acid of 1 mL, distilled water of 1 mL and ethanol of 7 mL [21]. Phase morphologies of the alloys were observed using scanning electron microscope (SEM, Vega III LMU/LMH, TESCAN, Brno, Czech Republic) equipment with an energy dispersive X-ray spectrometer (EDS). Microstructure analysis was performed through X-ray diffraction (XRD, Rigaku D/MAX-2500PC, Tokyo, Japan) with Cu Kα radiation.

### 2.3. Electrochemical Measurements

Electrochemical measurements were tested in 3.5 wt % NaCl solution at room temperature. Specimens were used as working electrodes and mounted in epoxy resin with an exposure area of 0.785 cm^2^ (Φ 10 mm). A platinum plate and a saturated calomel electrode were used as the counter and reference electrodes, respectively. Before testing, all the specimens were polished by using SiC sandpaper to 2000# followed by ultrasonic cleaning. Prior to potentiodynamic polarization testing, the open circuit potential of the alloys was measured for 20 min to establish an approximately steady state, followed by polarization curve measurement at a constant scan rate of 1 mV/s. Software CView (CView2, Wuhan CorrTest Co. Ltd., Wuhan, China) was used to fit the polarization curves. The electrochemical impedance spectroscopy (EIS) spectra were tested with the frequency range of 0.1–10^5^ Hz and a perturbation amplitude of 10 mV.

### 2.4. Immersion Tests

Samples with dimension of 10 × 10 × 3 mm were polished by SiC sandpaper from 120#–2000# and cleaned ultrasonically for immersion tests in 3.5 wt % NaCl solution at 25 ± 1 °C in an air-conditioned room. The ratio of surface area to solution volume was 1 cm^2^: 20 mL. The evolved hydrogen was collected into a burette above the samples [21]. It was monitored for about 37 h to evaluate the hydrogen evolution rate of the alloys in accordance with [21]. Immersion of 1 h and 98 h was conducted to observe the corrosion characteristics of samples. After 98 h immersion, the samples were removed from the solution, gently rinsed with distilled water, and dried in cold air. Corrosion products were removed using 200 g/L of chromic acid and 10 g/L of AgNO_3_ for 10 min. The weights loss were measured to calculate the corrosion rate following the ASTM-G31-72 [22]. The average corrosion rate CR (mm/y) was calculated according to the following equation [22]:

(1) CR=(K×W)/(A×T×D)

where K was a constant 8.76 × 10^4^, W was the weight loss (g), A was the surface area (cm^2^), T was the time exposed to solution (h), and D was the density of the material (g/cm^3^). For the immersion tests, three replicates were carried out for each condition to ensure the experimental reproducibility. The corrosion morphologies evolution of the specimens were also investigated by SEM with EDS.

## 3. Results and Discussion

### 3.1. Microstructural Analysis

**Figure 1:**
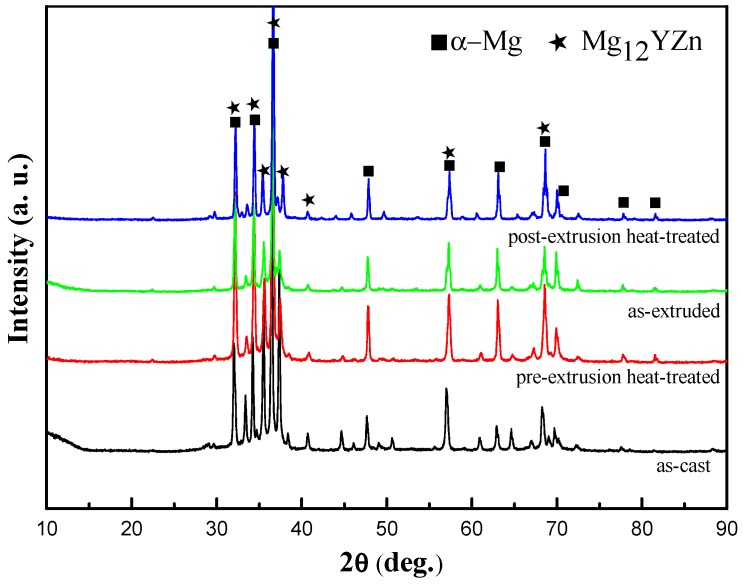
X-ray diffraction (XRD) patterns of Mg_95.33_Zn_2_Y_2.67_ alloy after different processes.

**Figure 2:**
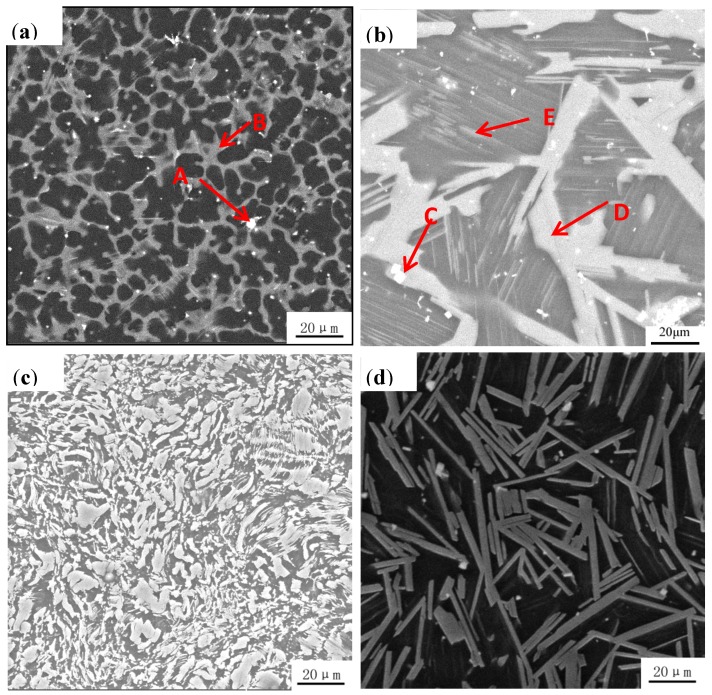
Scanning electron microscope (SEM) images of Mg_95.33_Zn_2_Y_2.67_ alloy with different morphologies of long-period stacking ordered (LPSO) phases: (**a**) as-cast, (**b**) pre-extrusion heat-treated, (**c**) as-extruded, (**d**) post-extrusion heat-treated.

**Figure 3:**
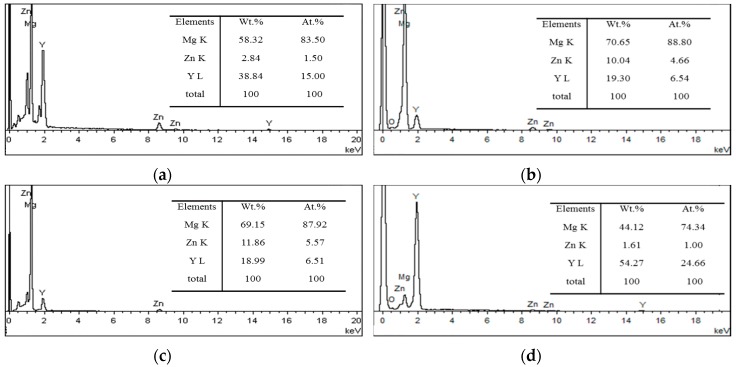
Energy dispersive X-ray spectrometer (EDS) results (**a**) Point A, (**b**) Point B, (**c**) Point C and (**d**) Point D for the main phases in as-cast and pre-extrusion heat-treated alloys.

The XRD result (Figure 1) of Mg_95.33_Zn_2_Y_2.67_ alloys after different processes are mainly composed of Mg and Mg_12_YZn, demonstrating that the second phases in all the current alloys are only LPSO phases. Although the phases are basically the same, the microstructure of these alloys changed significantly. Figure 2 presented a different morphology and distribution of LPSO phases in the alloys of different states: (a) as-cast, (b) pre-extrusion heat-treated, (c) as-extruded, (d) post-extrusion heat-treated. In the as-cast alloy (Figure 2a), the gray phase is the LPSO phase, and the black one is the Mg matrix. LPSO phases (indicated by B) in the as-cast alloys are connected together at the grain boundary in the block. A few white particles (indicated by A) dispersed could be confirmed as Y particles by the EDS results (Figure 3). After pre-extrusion heat treatment, compared with the as-cast alloys, the LPSO phases at the grain boundary are partially melted and become disconnected, part of which are gathered into a bigger block, like a rudimentary rod shape (indicated by C). In addition, a large number of lamellar LPSO phases (indicated by E) appears in the grain with larger white dots (indicated by D), which were also confirmed as Y particles by EDS (Figure 2b). When the alloy was extruded and crushed, the mass dispersion of different-sized particles are distributed irregularly in the matrix, as shown in Figure 2c. Part of them are extremely deformed, desultorily in a bent-strip shape. After heat treatment at 540 °C for 5 h followed by extrusion, a large number of relative regular, homogeneous rod LPSO phases are formed with a small amount of white dots (Figure 2d).

**Figure 4:**
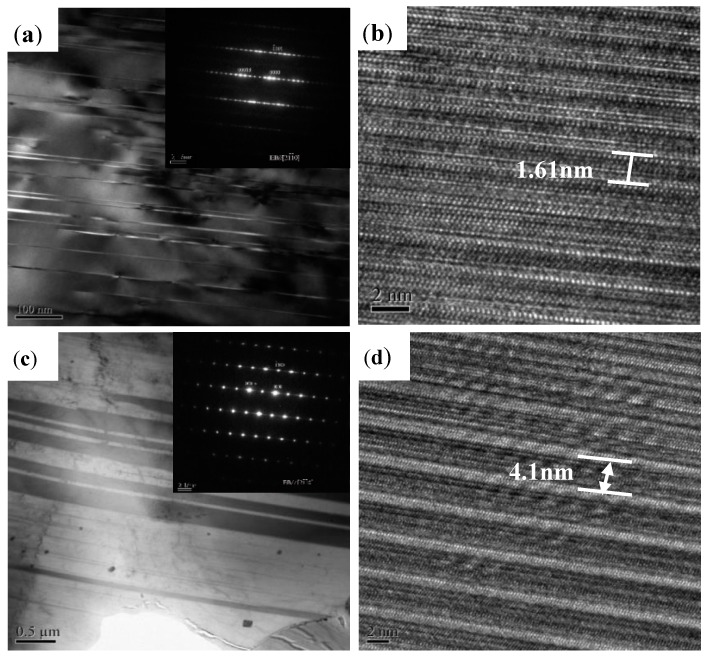
Bright-field transmission electron microscope (TEM) image of (**a**) block LPSO and (**c**) rod LPSO with corresponding selected area electron diffraction (SAED) patterns in the insets, respectively; (**b**,**d**) high-resolution electron microscope images (**a**,**c**) in Mg_95.33_Zn_2_Y_2.67_ alloy, respectively.

Figure 4 shows transmission electron microscopy (TEM) images of the LPSO phase of different shapes and its corresponding high-resolution image. Figure 4a shows a bright field TEM image of the block LPSO phase, which can be easily observed in the SEM image shown in Figure 2a, taken with the incident beam parallel to the [2$\bar{1}\bar{1}$0] direction of the hexagonal close-packed (HCP) Mg matrix. The diffraction spots are arranged asymmetrically with respect to the [0001] axis at positions that divide the distance between the incident-beam spot and the [0002] fundamental spot of the simple HCP unit cell into 18 parts. This arrangement indicates that the LPSO phase is made up of an 18-fold periodic stack of close-packed planes. In the high-resolution image (Figure 4b), the spacing between two similar lines is 1.61 nm, which also indicates that the LPSO phases obtained in the as-cast Mg–Zn–Y alloy are a 18R structure. As can be seen in Figure 4c, there is an obvious LPSO structure with additional diffraction spots in the LPSO structure with the magnesium matrix [0002], which can be divided into 14 equal parts. High resolution (Figure 4d) shows that the period length of LPSO is 4.1 nm, indicating that the rod-shaped LPSO phase is a 14H-type LPSO phase. In addition, the lamellar phase accompanied by the rod-shaped LPSO phase indicated by E was previously proved to be the same 14H-type LPSO phase as the rod-shaped one. The detailed evolution mechanism of this LPSO structure was also revealed systematically in our previous work [23,24].

In the present study, the alloys included mainly the LPSO phase and α-Mg. The as-cast alloys included 18R LPSO phase and 14H LPSO phase were obtained from the Mg-supersturated solid solution at high annealing temperature. After heat treatment at 540 °C for 5 h, the morphology of the gray block LPSO phase transformed into a regular and uniform gray rod-shaped phase, which is easy to form and difficult to dissolve.

### 3.2. Corrosion Behavior

**Figure 5:**
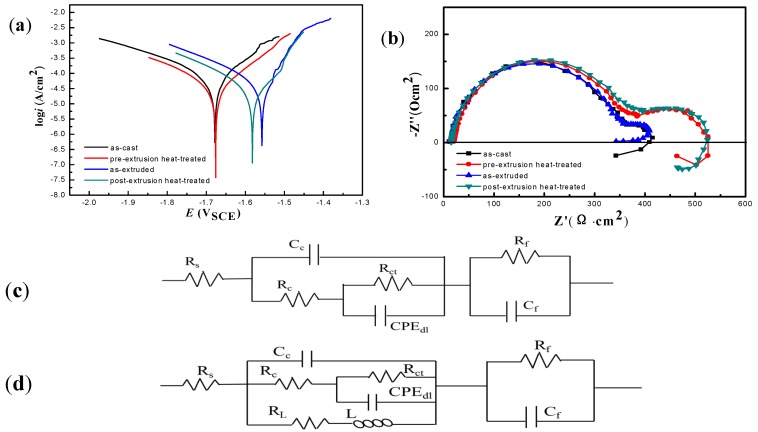
Potentiodynamic polarization curves (**a**) Nyquist plots (**b**) of Mg_95.33_Zn_2_Y_2.67_ alloy under different processes in 3.5 wt % NaCl solution; Equivalent circuit model (**c**) for the as-extruded and (**d**) for the rest three alloys.

Figure 5a shows the polarization curves of Mg_95.33_Zn_2_Y_2.67_ alloys with different LPSO phases in 3.5 wt % NaCl solution. They exhibit non-symmetrical curves and significantly differ from each other. The parameters of corrosion potential *E_corr_*, corrosion current density *i_corr_*, and polarization resistance *R_p_* fitted in *R_p_* mode are listed in Table 1. Compared with the as-cast alloy, both the anode and cathode current density of the pre-extrusion heat-treated alloy are decreased. Namely, heat treatment inhibits the cathode hydrogen evolution and weakens the anode dissolving due to the formation of rod-like LPSO phases. After extrusion, the cathode current density is increased, while the anode current density is decreased, implying the hydrogen evolution during cathode process is enhanced. The reason lies in the fact that fragmentation and deformation of LPSO phases cause a large increase in the number of microcells. The weakened anode reaction is attributed to the more uniform distribution of LPSO phases. It is well known that the uniform distribution of second phases could increase the randomness of galvanic corrosion, and make the corrosion occur uniformly, then decrease the anodic current density [25,26]. Compared with the extruded alloy, the anode current density of the post-extrusion heat-treated alloy is not changed, but the cathode current density is decreased. According to the corrosion parameters from Table 1, the rank of corrosion resistance of these alloys is listed as follows: the post-extrusion heat-treated > the pre-extrusion heat-treated > the as-extruded > the as-cast. The corrosion resistance of Mg_95.33_Zn_2_Y_2.67_ alloys has been improved to varying degrees after different process. Among them, the post-extrusion heat-treated one has improved the corrosion resistance to the maximum extent. In contrast to other alloys reported previously [9], such as as-cast Mg_97_Zn_1_Y_2_, as-extruded AZ31, as-cast WE43, as-cast ZK60 (Table 1), all the investigated alloys in the present work show a lower corrosion current density. Our previous work proved that the Mg_95.33_Zn_2_Y_2.67_ alloys exhibited good mechanical and damping properties [24]. Currently, it is revealed that the alloys present considerable anti-corrosion performance, indicating bright prospects for use of the present alloys in light structural materials.

Figure 5b shows the Nyquist curves of the different alloys. It exhibites a capacitive loop at high frequency and a capacitive loop at medium frequency. Only for the extruded alloy, the EIS curve is shown without an inductive arc, indicating that the alloy suffered no localized corrosion. The capacitive loop at high frequency represents the process of charge transfer in a galvanic corrosion, and its radius determines the corrosion resistance of the alloys. At the high-frequency region, the capacitive loop radius of the post-extrusion heat-treated alloy is the largest, suggesting the highest impedance value and the lowest corrosion rate. Meanwhile, with the processing changes, the capacitive loop radius decreases gradually. Moreover, the high-frequency capacitive reactance arcs for the extruded alloy and the as-cast alloy are basically in coincidence, which meant breakage and deformation of LPSO phases has few effects on the charge transfer process and the related corrosion resistance. It is worth mentioning that the distribution of LPSO phases in the extruded state alloy is more uniform, making the alloy more prone to uniform corrosion rather than localized corrosion.

The high frequency capacitive loops for the pre-extrusion heat-treated and the post-extrusion heat-treated alloys were slightly larger than those of the non-heat-treated alloys. But the diameter of the intermediate frequency capacitive arc was significantly increased. The capacitive loop in the high-frequency region was caused by the reaction between oxide film and corrosion medium. When Mg^2+^ reacted with OH^−^ to form Mg(OH)_2_, the barrier effect of the surface layer to the corrosion medium was fortified. However, the Cl^−^ continued to penetrate this oxide layer and reached the metal substrate due to its small volume and strong penetration ability in the corrosive medium. The capacitive loop at the medium frequency region was caused by the corrosion reaction of the metal substrate. Generally, the higher impedance value means the lower the corrosion rate. It was widely accepted that the arc of the intermediate frequency was related to the protective properties of the oxide film formed on the surface of the alloy [25,26,27]. The larger the aperture of the arc, the better the protective performance of the oxide film was, and the more it could inhibit the corrosion reaction. Therefore, the heat treatment could improve the protective properties of the oxide film significantly.

The equivalent circuit models constructed according to the Nyquist plots are shown in Figure 5c,d. Figure 5c was fitted for the as-extruded alloy and Figure 5d was fitted for the other three alloys. The corresponding fitted parameters are listed in Table 2, from which it is easy to find that the post-extrusion heat-treated alloy has the largest corrosion resistance, which verified the conclusions by the polarization curves further.

**Figure 6:**
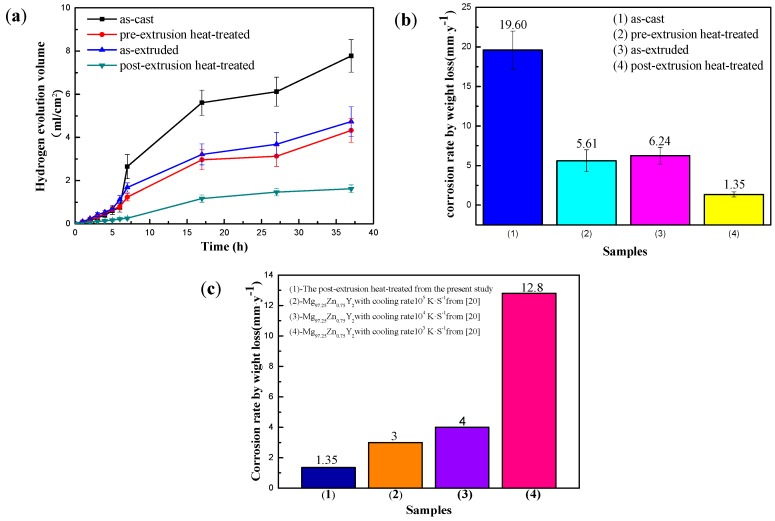
Hydrogen evolution curves for 37 h (**a**) weight-loss corrosion rate for 98 h; (**b**) of Mg_95.33_Zn_2_Y_2.67_ alloy by immersing in 3.5 wt % NaCl solution; (**c**) corrosion rate of the post-extrusion heat-treated alloy with other alloys reported [27].

Figure 6a presents the corrosion behavior of Mg_95.33_Zn_2_Y_2.67_ alloys characterized by hydrogen evolution. For each specimen, there is an incubation period with a low rate of hydrogen evolution. After that, the rate of hydrogen evolution is substantially greater, typically tending to a steady state. The incubation period is the longest for the post-extrusion heated alloy and shortest for the as-cast alloy. Thus, the corrosion behavior is reasonably characterized by an initial period of low corrosion rate during an incubation period, followed by a period of increasing corrosion rate, and subsequently steady state corrosion. The overall extent of the corrosion attack is ranked as: the post-extrusion heat-treated < the pre-extrusion heated < the as-extruded < the as-cast. Figure 6b shows the average corrosion rates of Mg_95.33_Zn_2_Y_2.67_ alloys under different conditions evaluated by the weight loss method. Among these alloys, the post-extrusion heated one shows the lowest corrosion rate, while the as-cast one shows the highest, which indicates that the corrosion rates of all the Mg_95.33_Zn_2_Y_2.67_ alloys are also in the same order as the result of the hydrogen evolution test. Compared to the reported Mg_97.25_Zn_0.25_Y_2_ alloy with the LPSO phase [27], the post-extrusion heat-treated alloy of our work presented a much lower corrosion rate, as shown in Figure 6c, indicating the formation of the rod-shaped LPSO phase is an effective way to enhance the corrosion property of Mg–Zn–Y alloys.

### 3.3. Corroded Surface Evaluation

**Figure 7:**
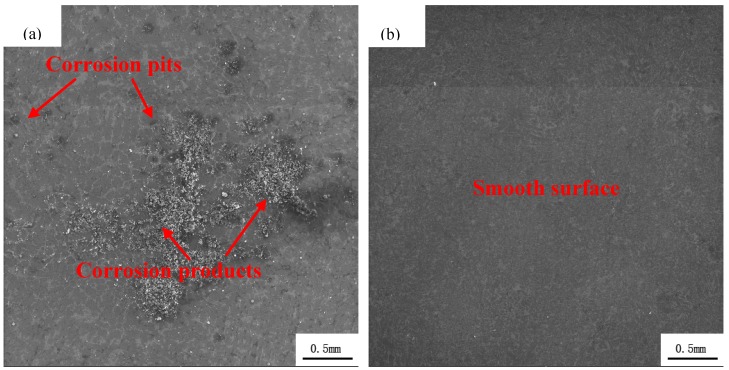
Corrosion morphologies of as-cast (**a**) and as-extruded (**b**) Mg_95.33_Zn_2_Y_2.67_ alloy after the electrochemical impedance spectroscopy (EIS) test.

The morphological surface of as-cast (a) and as-extruded (b) Mg_95.33_Zn_2_Y_2.67_ alloys after EIS tests is shown in Figure 7. Lots of obvious corrosion pits appear on the as-cast one (indicated by the red arrows), while the surface of the as-extruded one is smooth without visible pits. This is because extrusion broke the LPSO phases into uniformly distributed fragments in the alloy, relieving the localized corrosion. 

**Figure 8:**
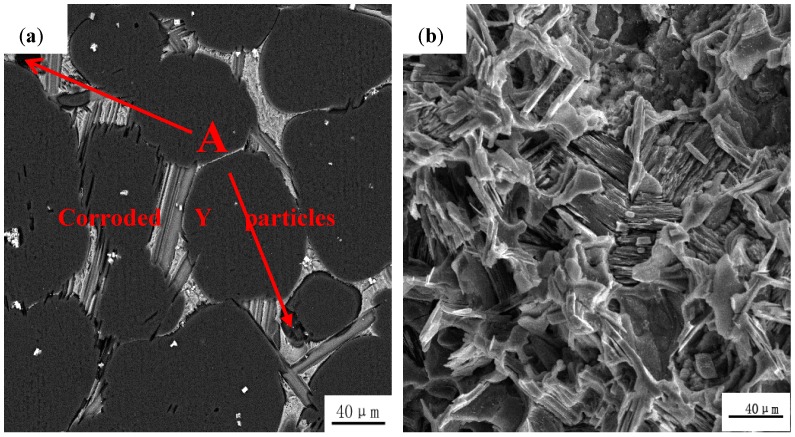
Corrosion morphologies of pre-extrusion heat-treated Mg_95.33_Zn_2_Y_2.67_ alloy after removing the corrosion products for 1 h (**a**) and 98 h (**b**) immersion.

In order to further clarify the reason why the corrosion resistance of pre-extrusion heat treatment alloys is higher, short- and long-term immersion tests were carried out. As shown in Figure 8, after the short-term immersion of 1 h, a small number of corrosion pits appears on the magnesium matrix, and part of the Y particles are corroded. However, after long-term immersion of 98 h, only the gray LPSO phases existed in the alloy, suggesting that all the Y particles were corroded and peeled off from the Mg matrix. As previously reported [14,28], the cathode activity of the Y particles is more positive than the Mg matrix. The areas around the Y particles are the initiating sites of the galvanic corrosion between the Y particles and the Mg matrix. With the corrosion proceeding, the areas around the Y particles were basically dissolved and the Y particles gradually fell from the matrix. Then the rudimentary rod-shaped LPSO phases were exposed directly in the medium. The appearance of the rod-shaped prototype LPSO phases could act as a physical barrier, preventing the corrosive medium from penetrating into the substrate. Thus, the Mg dissolution reaction is hindered and the corrosion resistance has been improved to some extent.

**Figure 9:**
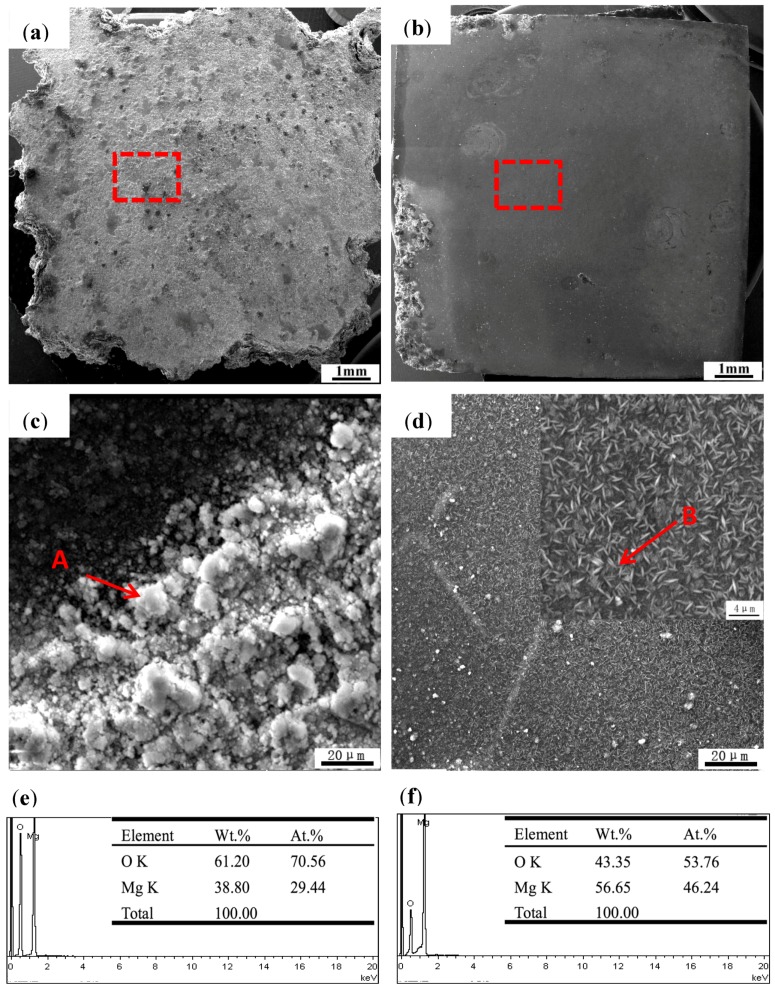
Surface morphologies of macrograph (**a**), micrograph (**c**) (red square in a) of as-extruded; macrograph (**b**), micrograph (**d**) (red square in b) of post-extrusion heat-treated Mg_95.33_Zn_2_Y_2.67_ alloys after 98 h immersion; EDS analysis results of point A (**e**) and point B (**f**).

The corrosion morphologies of the extruded and post-extrusion heat-treated alloys are compared in Figure 9. After 98 h immersion, the macro-surface of the extruded alloy and the post-extrusion heat-treated alloy are apparently different, as shown in Figure 9a,b, respectively. The former is basically corroded with a rough surface, while the latter exhibits a smooth surface with a little corroded area. Furthermore, the corrosion surface for the extruded alloy is covered with plentiful circular-porous products marked with A, which are mainly composed of 70.56 at % O and 29.44 at % Mg by EDS analysis, as shown in Figure 9c. However, the post-extrusion heat-treated alloy surface is covered with very thin and uniform corrosion products, as shown in Figure 9d. From the amplified inset, products like thin strips are distributed out of order. The EDS results indicate the main components are still O and Mg, with a content of 53.76 at % and 46.24 at %, respectively. It is notable that the main compositions of the corrosion products are the same, but the circular products with a high content of O imply a relatively loose and porous structure, and the strip products with a low content of O are compact, dense and uniform.

The kinetics of magnesium corrosion can be attributed to the properties of the surface film. The formation of Mg(OH)_2_ film on the magnesium surface is relatively stable compared to other magnesium compounds. However, the existence of strong corrosive ions in aqueous solution, such as Cl^−^, could severely damage the magnesium surface film. According to the EIS spectrum from Figure 5b, the pore diameter of the intermediate frequency arc of the post-extrusion heat-treated alloy is obviously greater than that of the extruded state alloy, implying the best protective properties of the oxide film formed on the surface. For the post-extrusion heat-treated alloy, the LPSO phases are of different lengths without orientation distribution, as shown in Figure 2d. It was proposed by [28,29] that the LPSO phases were the effective cathode phases in the Mg–Zn–Y alloy, and corrosion generally occurred in the magnesium matrix around the LPSO phases. In this study, since LPSO phases in the post-extrusion heat-treated alloys are of regular and uniform rod shape, corrosion initiated at the adjacent region of the LPSO phases. With the corrosion proceeding, the corrosion products formed along the orientation of rods, resulting in the corrosion products of a long strip rather than being round. Thus, it is easy to understand the extrusion and heat treatment mainly changed the morphology and distribution of the LPSO phases, and then caused the formation of thin-strip corrosion products. These dense and fine strips significantly increased the compactness of the oxide film and enhanced the protective property of the membrane. Furthermore, this rod-shaped LPSO phase is hard to dissolve and could act as a barrier to resist the corrosive ions. Finally, the protective film of corrosion products and the barrier effect of the rod-shaped LPSO phases are combined to influence the corrosion property.

**Figure 10:**
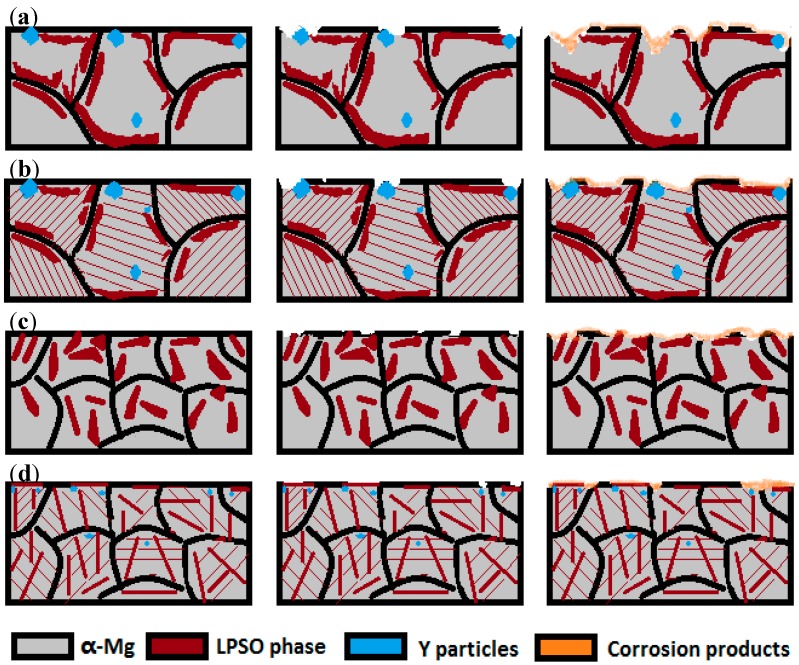
Schematic map during corrosion process for as-cast (**a**); pre-extrusion heat-treated (**b**); as-extruded (**c**); and post-extrusion heat-treated; (**d**) Mg_95.33_Zn_2_Y_2.67_ alloy.

Figure 10 shows a schematic map during the corrosion process for Mg_95.33_Zn_2_Y_2.67_ alloys under different technical processes. For the as-cast alloy, galvanic corrosion occurs at the Mg matrix around the big LPSO blocks and develops into large and deep holes, which is typical of localized corrosion (Figure 10a). The LPSO blocks turn into a rudimentary rod-shaped structure for the pre-extrusion heated one, which could act as a barrier to hinder the penetration of Cl^−^ (Figure 10b), and improve the corrosion performance to some extent. After extrusion, the LPSO phases are broken into uniformly distributed fragments in the alloy, leading to a more uniform corrosion without localized holes (Figure 10c). That illustrates well why the as-extruded alloy has a higher corrosion resistance than the as-cast alloy. As for the post-extrusion heat-treated one, the rod-shaped LPSO phases lead to the formation of dense and long-strip corrosion products, which are compact to resist the attack of the corrosive ions (Figure 10d). Moreover, the rod-shaped LPSO phases themselves could also act as a barrier to retard the corrosion progress. Thus, these two combined effects enhance the corrosion performance of the post-extrusion heated alloy to a maximum. Our work demonstrates that the improved corrosion resistance of magnesium alloys with LPSO can be tailored effectively by the preceding technology and post-heat treatment.

## 4. Conclusions

The morphological evolution of LPSO phases in the Mg_95.33_Zn_2_Y_2.67_ alloys under as-cast, pre-extrusion heat treatment, extrusion, post-extrusion heat treatment can be described as: block LPSO phases with few Y particles → rudimentary rod-shaped LPSO phases with bigger Y phases → fine and deformed fragments of the LPSO phases → regular and uniform rod-shaped LPSO phases with a few Y phases. Their corresponding corrosion resistances are improved to varying degrees and the rank is stated as: post-extrusion heat-treated > pre-extrusion heat-treated > as-extruded > as-cast. Pre-extrusion heat treatment reduced the integral cathode activity and anode dissolution due to the appearance of rudimentary rod-shaped LPSO phases. Extrusion could make the corrosion more uniform because of evenly distributed second phases. The post-extrusion heat treatment caused rod-shaped LPSO phases, which led to the formation of fine-strip corrosion film and prevented the corrosive ions penetrating further effectively. Thus, the corrosion resistance for the post-extrusion heat treatment of Mg_95.33_Zn_2_Y_2.67_ alloy has been improved to the greatest extent.

## Figures and Tables

**Table 1:** Parameters of potentiodynamic polarization curves fitted by *R_p_* mode and the typical alloys previously reported.

Alloy	*E_corr_* (V)	*i_corr_* (A/cm^2^)	*R_p_*	
as-cast	−1.6792	5.4 × 10^−5^	480	Present work
pre-extrusion heat-treated	−1.6768	3.40 × 10^−5^	770.7	Present work
as-extruded	−1.5582	4.4 × 10^−5^	586.6	Present work
post-extrusion heat-treated	−1.5823	2.9 × 10^−5^	905.2	Present work
as-cast Mg_97_Zn_1_Y_2_	−1.488	7.6 × 10^−5^	-	[9]
as-extruded AZ31	−1.518	8.7 × 10^−5^	-	[9]
as-cast WE43	−1.69	5.9 × 10^−5^	-	[9]
as-cast ZK60	−1.544	2.8 × 10^−4^	-	[9]

**Table 2:** The fitted parameters of the equivalent circuit model by the Nyquist plots.

Parameters	As-Cast	Pre-Extrusion Heat Treated	As-Extruded	Post-Extrusion Heat Treated
*R_s_* (Ω·cm^2^)	8.68	16.52	8.857	13.36
*R_c_* (Ω·cm^2^)	4.74	4.27	5.50	0.098
*C_c_* (F·cm^2^)	3.38 × 10^−7^	2.32 × 10^−7^	2.90 × 10^−7^	1.85 × 10^−13^
*CPE-T* (μΩ^−1^·cm^−2^·s^−1^)	2.55 × 10^−5^	2.32 × 10^−5^	3.00 × 10^−5^	2.18 × 10^−5^
*CPE-P*	0.91279	0.90695	0.91894	0.91298
*R_ct_* (Ω·cm^2^)	339.8	350.8	343	548.2
*R_L_* (Ω·cm^2^)	1110	640.6	-	1716
*L* (H·cm^2^)	9989	9261	-	22,845
*R_f_* (Ω·cm^2^)	63.3	153.2	50.3	133.7
*C_f_* (F·cm^2^)	0.0074	0.0038	0.0073	0.0034
*chisq*	0.0020	0.0014	0.0033	0.0011

## References

[1] Zhang L., Zhou W., Hu P.H., Zhou Q. (2016). Microstructural characteristics and mechanical properties of Mg–Zn–Y alloy containing icosahedral quasicrystals phase treated by pulsed magnetic field. J. Alloys Compd..

[2] Asgharzadeh H., Yoon E.Y., Chae H.J., Kim T.S., Lee J.W., Kim H.S. (2014). Microstructure and mechanical properties of a Mg–Zn–Y alloy produced by a powder metallurgy route. J. Alloys Compd..

[3] Kawamura Y., Hayashi K., Inoue A., Masumoto T. (2001). Rapidly solidified powder metallurgy Mg_97_Zn_1_Y_2_ Alloys with excellent tensile yield strength above 600 MPa. Mater. Trans..

[4] Rosalie J.M., Somekawa H., Singh A., Mukai T. (2013). Effect of precipitation on strength and ductility in a Mg–Zn–Y alloy. J. Alloys Compd..

[5] Jiang H., Qiao X., Xu C., Kamado S., Wu K. (2018). Influence of size and distribution of W phase on strength and ductility of high strength Mg-5.1Zn-3.2Y-0.4Zr-0.4Ca alloy processed by indirect extrusion. J. Mater. Sci. Technol..

[6] Wang W., Xu R., Hao Y., Wang Q., Wang Q., Yu L., Che Q., Cai J., Wang K., Ma Z. (2018). Corrosion fatigue behavior of friction stir processed interstitial free steel. J. Mater. Sci. Technol..

[7] Miao H., Huang H., Shi Y., Zhang H., Pei J., Yuan G. (2017). Effects of solution treatment before extrusion on the microstructure, mechanical properties and corrosion of Mg–Zn–Gd alloy in vitro. Corros. Sci..

[8] Zhao X., Shi L., Xu J. (2013). Mg–Zn–Y alloys with long-period stacking ordered structure: In vitro assessments of biodegradation behavior. Mater. Sci. Eng. C.

[9] Song Y., Shan D., Chen R., Han E.H. (2010). Effect of second phases on the corrosion behaviour of wrought Mg–Zn–Y–Zr alloy. Corros. Sci..

[10] Wang J.F., Jiang W.Y., Ma Y., Li Y., Huang S. (2018). Substantial corrosion resistance improvement in heat-treated Mg–Gd–Zn alloys with a long period stacking ordered structure. Mater. Chem. Phys..

[11] Grobner J., Kozlov A., Schmid-Fetzer R. (2012). Phase equilibria and transformations in ternary Mg-rich Mg–Y–Zn alloys. Acta Mater..

[12] Kawamura Y., Yamasaki M. (2007). Formation and Mechanical Properties of Mg97Zn1RE2 Alloys with Long-Period Stacking Ordered Structure. Mater. Trans..

[13] Zhang X., Wu Y., Xue Y., Wang Z., Yang L. (2012). Biocorrosion behavior and cytotoxicity of a Mg–Gd–Zn–Zr alloy with long period stacking ordered structure. Mater. Lett..

[14] Zhang X., Ba Z., Wang Q., Wu Y., Wang Z., Wang Q. (2014). Uniform corrosion behavior of GZ51K alloy with long period stacking ordered structure for biomedical application. Corros. Sci..

[15] Li C.Q., Xu D.K., Zeng Z.R., Wang B.J., Sheng L.Y., Chen X.B., Han E.H. (2017). Effect of volume fraction of LPSO phases on corrosion and mechanical properties of Mg–Zn–Y alloys. Mater. Des..

[16] Izumi S., Yamasaki M., Kawamura Y. (2009). Relation between corrosion behavior and microstructure of Mg–Zn–Y alloys prepared by rapid solidification at various cooling rates. Corros. Sci..

[17] Pérez P., Onofre E., Caheza S., Llorente I., del Valle J.A., GarcÍa-Alonso M.C., Adeva P., Escudero M.L. (2013). Corrosion behaviour of Mg–Zn–Y–Mischmetal alloys in phosphate buffer saline solution. Corros. Sci..

[18] Wang S.D., Xu D.K., Chen X.B., Han E.H., Dong C. (2015). Effect of heat treatment on the corrosion resistance and mechanical properties of an as-forged Mg–Zn–Y–Zr alloy. Corros. Sci..

[19] Song Y., Han E.H., Shan D., Yim C.D., You B.S. (2012). The effect of Zn concentration on the corrosion behavior of Mg–xZn alloys. Corros. Sci..

[20] Song G.L., Atrens A., StJohn D. (2001). An hydrogen evolution method for the estimation of the corrosion rate of magnesium alloys. Magnes. Technol..

[21] Abidin N.I.Z., Atrens A.D., Martin D., Atrens A. (2011). Corrosion of high purity Mg, Mg2Zn0.2Mn, ZE41 and AZ91 in Hank’s solution at 37 °C. Corros. Sci..

[22] ASTM-G31-72 (2004). Standard Practice for Laboratory Immersion Corrosion Testing of Metals.

[23] Wang J., Song P., Zhou X., Huang X., Pan F. (2012). Influence of the morphology of long-period stacking ordered phase on the mechanical properties of as-extruded Mg-5Zn-5Y-0.6Zr magnesium alloy. Mater. Sci. Eng. A.

[24] Lu R., Wang J., Chen Y., Qin D., Yang W. (2015). Effects of heat treatment on the morphology of long-period stacking ordered phase, the corresponding damping capacities and mechanical properties of Mg–Zn–Y alloys. J. Alloys Compd..

[25] Mandal M., Moon A.P., Deo G., Mendis C.L., Mondal K. (2014). Corrosion behavior of Mg–2.4Zn alloy micro-alloyed with Ag and Ca. Corros. Sci..

[26] Song G. (2009). Effect of tin modification on corrosion of AM70 magnesium alloy. Corros. Sci..

[27] Yamasaki M., Izumi S., Kawamura Y., Habazaki H. (2011). Corrosion and passivation behavior of Mg–Zn–Y–Al alloys prepared by cooling rate-controlled solidification. Appl. Surf. Sci..

[28] Zhang J., Xu J., Cheng W., Chen C., Kang J. (2012). Corrosion Behavior of Mg–Zn–Y alloy with Long-period stacking ordered structure. J. Mater. Sci. Technol..

[29] Zhang X., Kairy S.K., Dia J., Birbilis N. (2018). A Closer Look at the Role of Nanometer Scale Solute-Rich Stacking Faults in the Localized Corrosion of a Magnesium Alloy GZ31K. J. Electrochem. Soc..

